# Supplementary material for: Coherent X-rays reveal anomalous molecular diffusion and cage effects in crowded protein solutions
Source: Nat Commun. 2025 Nov 29;16:10814. doi: 10.1038/s41467-025-66972-6 (PMC12669764; doi:10.1038/s41467-025-66972-6)
Supplement: Supplementary file 1 — Supplementary information [file 41467_2025_66972_MOESM1_ESM.pdf]

Supplementary Information:

Coherent X-rays reveal anomalous molecular diffusion and cage effects in crowded protein solutions

Anita Girelli[1,2,*], Maddalena Bin[1], Mariia Filianina[1], Michelle Dargasz[3], Nimmi Das Anthuparambil[3], Johannes Möller[4], Alexey Zozulya[4], Iason Andronis[1], Sonja Timmermann[3], Sharon Berkowicz[1], Sebastian Retzbach[2], Mario Reiser[1], Agha Mohammad Raza[3], Marvin Kowalski[3], Mohammad Sayed Akhundzadeh[3], Jenny Schrage[3], Chang Hee Woo[10], Maximilian D. Senft[2], Lara Franziska Reichart[2], Aliaksandr Leonau[3], Prince Prabhu Rajaiah[5,6], William Chèvremont[7], Tilo Seydel[8], Jörg Hallmann[4], Angel Rodriguez-Fernandez[4], Jan-Etienne Pudell[4], Felix Brausse[4], Ulrike Boesenberg[4], James Wrigley[4], Mohamed Youssef[4], Wei Lu[4], Wonhyuk Jo[4], Roman Shayduk[4], Anders Madsen[4], Felix Lehmkühler[5,9], Michael Paulus[10], Fajun Zhang[2], Frank Schreiber[2], Christian Gutt[3], and Fivos Perakis[1,*]

[1]Department of Physics, AlbaNova University Center, Stockholm University, 10691 Stockholm, Sweden
[2]Institut für Angewandte Physik, Universität Tübingen, Auf der Morgenstelle 10, 72076 Tübingen, Germany
[3]Department Physik, Universität Siegen, Walter-Flex-Strasse 3, 57072 Siegen, Germany
[4]European X-Ray Free-Electron Laser Facility, Holzkoppel 4, 22869 Schenefeld, Germany
[5]The Hamburg Centre for Ultrafast Imaging, Luruper Chaussee 149, 22761 Hamburg, Germany
[6]Institute for Biochemistry and Molecular Biology, Laboratory for Structural Biology of Infection and Inflammation, University of Hamburg, c/o DESY, 22603, Hamburg, Germany
[7]ESRF - The European Synchrotron, 71 Avenue des Martyrs, 38042 Grenoble, France
[8]Institut Laue-Langevin, 71 Avenue des Martyrs, 38042 Grenoble, France
[9]Deutsches Elektronen-Synchrotron DESY, Notkestr. 85, 22607 Hamburg, Germany
[10]Fakultät Physik/DELTA, TU Dortmund, 44221 Dortmund, Germany
[*]Email: anita.girelli@fysik.su.se, f.perakis@fysik.su.se

Table of contents

1 **Data filtering** 2

2 **Structure factor interpolation** 2

3 **Assessment of beam-induced effects** 3

4 **Estimation of protein diffusion coefficient in the dilute limit** 7

5 $H(q)$ **model fit** 7

6 **Contrast evaluation** 8

1 Data filtering

To achieve a sufficient signal-to-noise ratio (SNR), the measurements were repeated multiple times, including different sample positions, capillaries and batches of samples. Two filtering criteria were applied to ensure data quality, based on (1) the sample position and (2) the two-time correlation (TTC) functions. The first filter excluded measurements where the capillary was only partially hit or air bubbles were present. For the second filter we computed the average value and the standard deviation on a single train basis. The parameters chosen for the calculation of the correlation functions are shown in Table S1, including the protein concentration, c, number of pulses per train, n_{pulses}, number of repetitions, n_{rep}, incident X-ray transmission, T_0, and corresponding fluence.

Table S1 | XPCS parameters for the calculation of the $g_2(q,t)$

c (mg/ml)	n_{pulses}	n_{rep}	T_0	Fluence (nJ/μm^2/pulse)
70	120	5000	5.35×10^{-6}	7.14
180	120	5000	5.35×10^{-6}	7.19
400	120	5000	5.35×10^{-6}	7.27
730	120	5000	5.35×10^{-6}	7.41

2 Structure factor interpolation

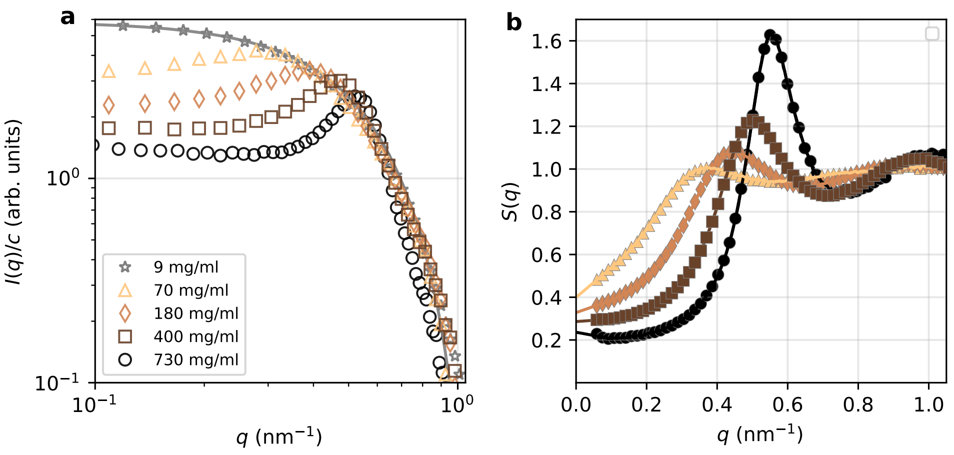

Fig. S1 | (a) Scattering intensity, $I(q)$, from the first pulse per train for various concentrations c and transmission value $T_0 = 5.35 \times 10^{-6}$. The solid line is a fit using the spherical form factor. (b) The experimental data of structure factor, $S(q)$, at various concentrations (as indicated by the symbols in the legend of panel a) and their interpolated values (solid lines).

In Fig. S1a is shown the scattering intensity, $I(q)$, as reported in the main manuscript, but in a log-log scale. The corresponding structure factor, $S(q)$, was determined by ensuring that its values at high q oscillated around 1. The validity of the normalization was checked by comparing the data with those obtained at ESRF (see main manuscript), which allowed for measurements at higher q values. The experimental $S(q)$ data and their interpolated values used for the calculation of the $\delta\gamma$ theory are shown in Fig.S1b. To ensure reliable results, we included the values $S(\infty) = 1$ and $S(0) = C_{lin}S(q_{min})$, where C_{lin} is the intercept from a linear fit of the $S(q)$ in a q-range between 0.07 and 0.125 nm^{-1}.

3 Assessment of beam-induced effects

Establishing optimal measurement conditions, where both structural and dynamic properties are not affected by the X-ray beam, is crucial for XPCS. Following approaches of previous studies [1], we monitored changes in both the scattering intensity, $I(q)$, and diffusion coefficient, $D(q)$.

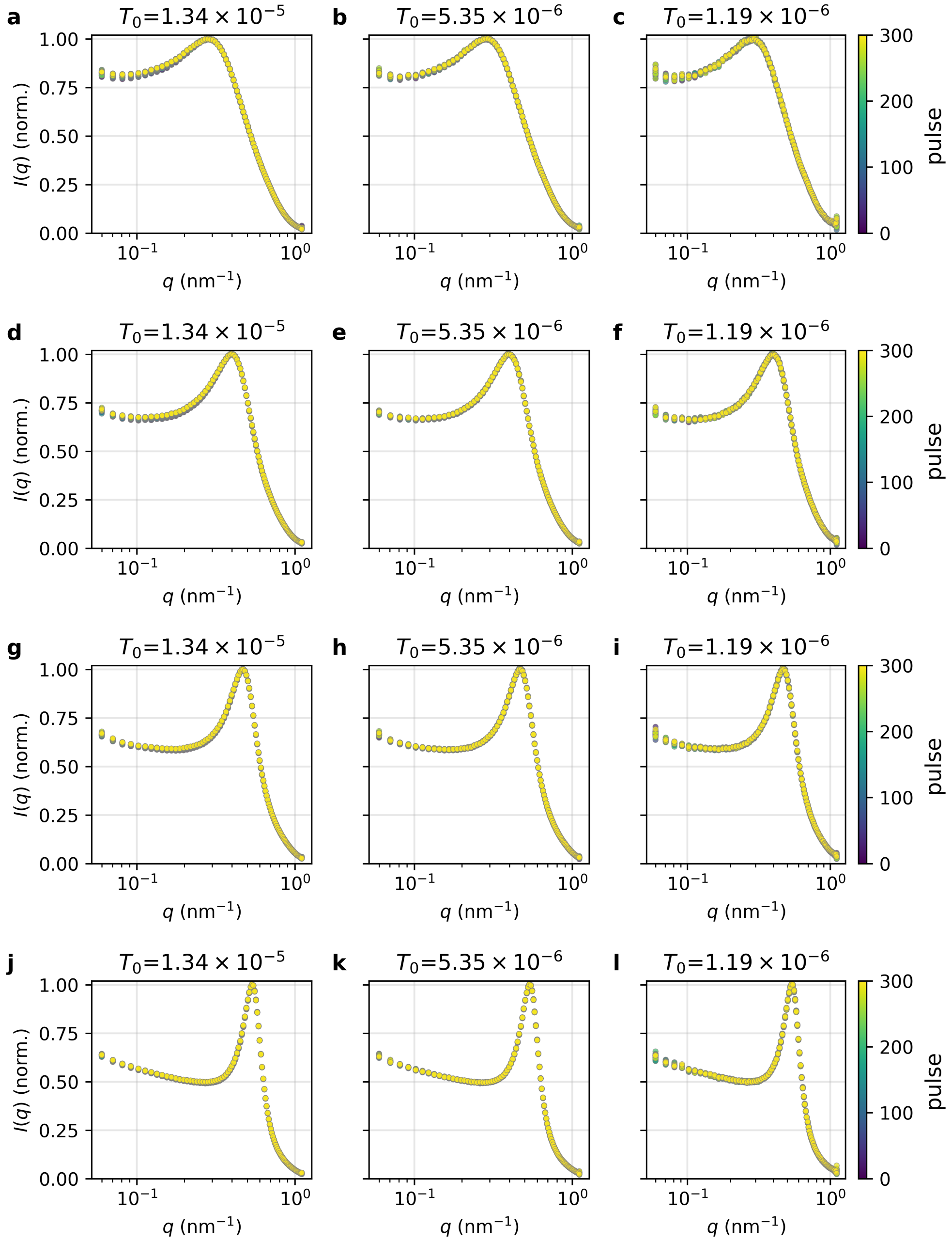

Fig. S2 | Scattering intensity, $I(q)$, normalised by the intensity value at the peak position q_0 at different transmission values T_0, and concentrations $c = 70$ mg/ml (a-c), $c = 180$ mg/ml (d-f), $c = 400$ mg/ml (g-i) and $c = 730$ mg/ml (j-l). The colors correspond to different pulse numbers as indicated in the color bar.

In Fig. S2, the $I(q)$ at different transmission values, T_0, and concentrations, c, is shown. The only visible changes in the intensity involve a subtle increase in the small q region ($q < 0.4$ nm^{-1}). To quantify this change, we calculate the mean scattering intensity over 0.1 nm^{-1} $< q <$ 0.3 nm^{-1}, normalised to the intensity value of the first pulse, which is shown in Fig.S3. A linear increase of the signal as a function of train pulse number, n_{pulse}, was observed for all concentrations c and transmission values T_0. Under the measurements conditions presented in the main paper ($n_{pulse} = 120$, $T_0 = 5.35 \times 10^{-6}$), the relative changes were less than 0.5%.

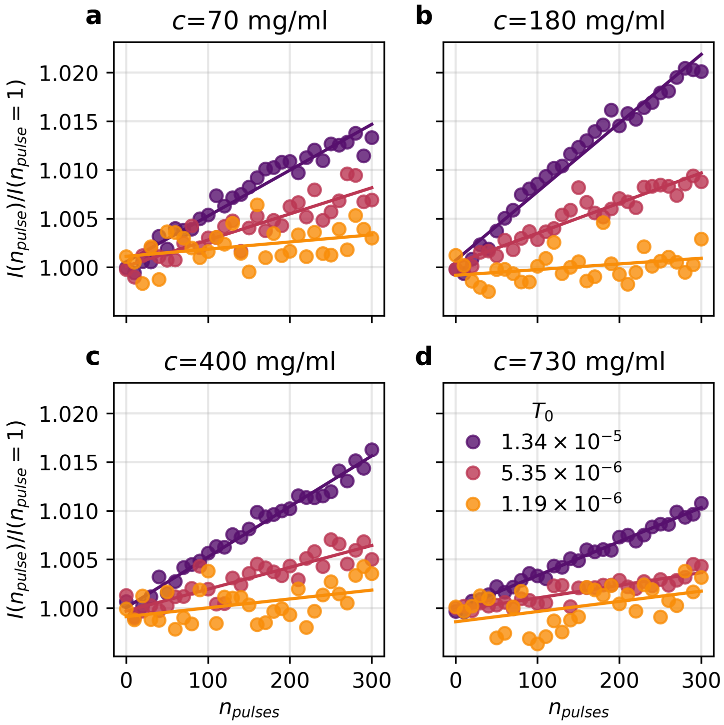

Fig. S3 | The scattering intensity, $I(q)$, averaged over the range 0.1 nm^{-1} $< q <$ 0.3 nm^{-1}, normalized by the scattering intensity at the first pulse, $I(n_{pulse} = 1)$, as a function of pulse number n_{pulse} for the various transmission values, T_0., and concentrations, $c = 70$ mg/ml (a), $c = 180$ mg/ml (b), $c = 400$ mg/ml (c) and $c = 730$ mg/ml (d).

From the TTC maps shown in Fig.S4, it is possible to quantify possible beam-induced effects on dynamics. The data reveal no noticeable changes in the width along the diagonal. This observation, combined with the changes below 0.5% seen in the $I(q)$, suggests that protein aggregation is not detected within the timescale probed.

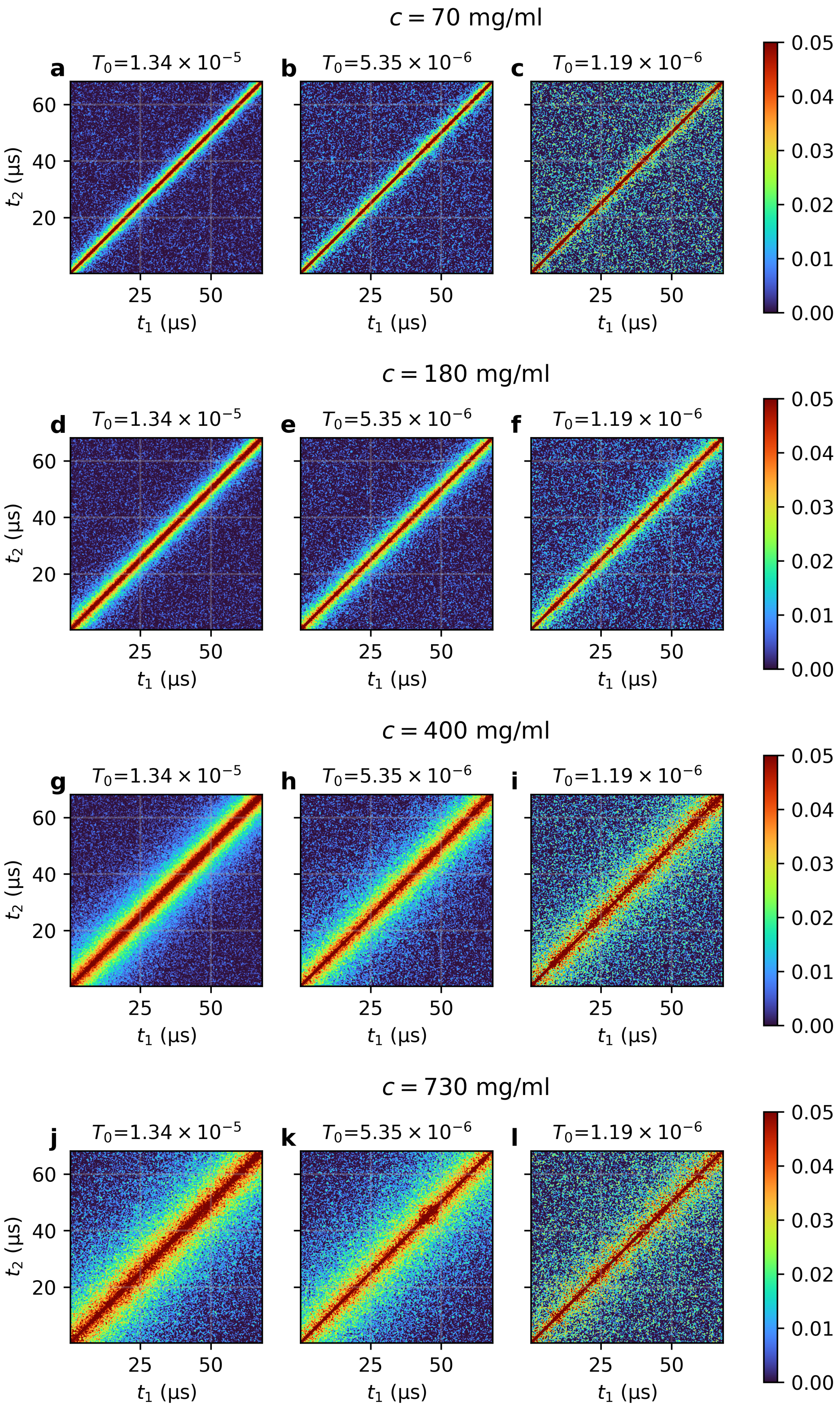

Fig. S4 | TTCs at $0.275\ \mathrm{nm}^{-1} < q < 0.325\ \mathrm{nm}^{-1}$ measured at 25° C for a protein concentration of (a-c) 70 mg/ml, (d-f) 180 mg/ml, (g-i) 400 mg/ml, (j-l) 730 mg/ml, with different beam transmissions reported on the top of each panel.

More subtle X-ray beam-induced effects can be quantified by examining the changes of the intensity autocorrelation function g_2 line shape. To compute the g_2 functions, 100 pulses were used and the initial and final pulse were varied to track possible changes in the diffusion as function of pulse number. Each g_2 curve was associated with a pulse number, which is the average of the first and the last pulse numbers. The correlation functions were fitted with a stretched exponential function, as in the main manuscript. Figure S5a shows the resulting diffusion coefficients, averaged over $0.275\ \mathrm{nm}^{-1} < q < 0.375\ \mathrm{nm}^{-1}$, as a function of pulse number. We attribute the observed changes to beam-induced heating [1, 2]. The temperature increase is quantified by the rise in the value of the diffusion coefficient using Stokes-Einstein equation, $D(T+\Delta T) = k_b(T+\Delta T)/[6\pi\eta(T+\Delta T)R]$, by accounting for the increase in diffusion coefficient and change in viscosity. The estimated temperature rise is shown in Table S2. The corresponding Kohlrausch-Williams-Watts (KWW) exponent, α, is shown in Fig S5b, where α remains nearly constant as a function of pulse number.

Table S2 | Beam-induced temperature increase estimated for the parameters used for the calculation of the correlation functions shown in Fig.3 (in the main paper). The parameters used are $T_0 = 5.35 \times 10^{-6}$ and $n_{pulses} = 120$.

c (mg/ml)	ΔT (K)
70	0.95 ± 0.11
180	0.65 ± 0.09
400	1.07 ± 0.07
730	0.85 ± 0.11

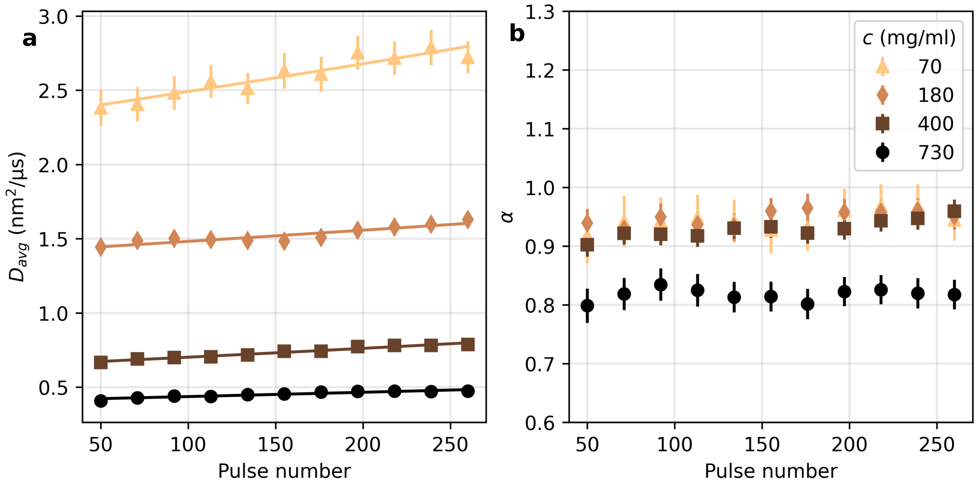

Fig. S5 | (a) The diffusion coefficient, averaged over $0.275\ \mathrm{nm}^{-1} < q < 0.375\ \mathrm{nm}^{-1}$, as a function of pulse number for different concentrations. The solid line represent the fitted increase of diffusion coefficient due to temperature increase. (b) The Kohlrausch-Williams-Watts (KWW) exponent, α, as a function of pulse number for different concentrations.

4 Estimation of protein diffusion coefficient in the dilute limit

The protein diffusion coefficient, D_0, in the dilute limit and the corresponding hydrodynamic radius R_h were estimated with DLS by measuring a solution with $c = 9$ mg/ml in water-glycerol (55 vol% glycerol). The measurement was performed with a LS spectrometer (LS Instruments AG) equipped with a λ = 660 nm CW laser. The computed g_2 functions are reported in Fig.S6a. The decorrelation rate $\Gamma(q)$ was extracted by fitting a single exponential decay, with the expression $g_2(q,t) = \beta(q)\exp[-2t\Gamma(q)]$. The extracted decorrelation rates are shown in Fig. S6b.

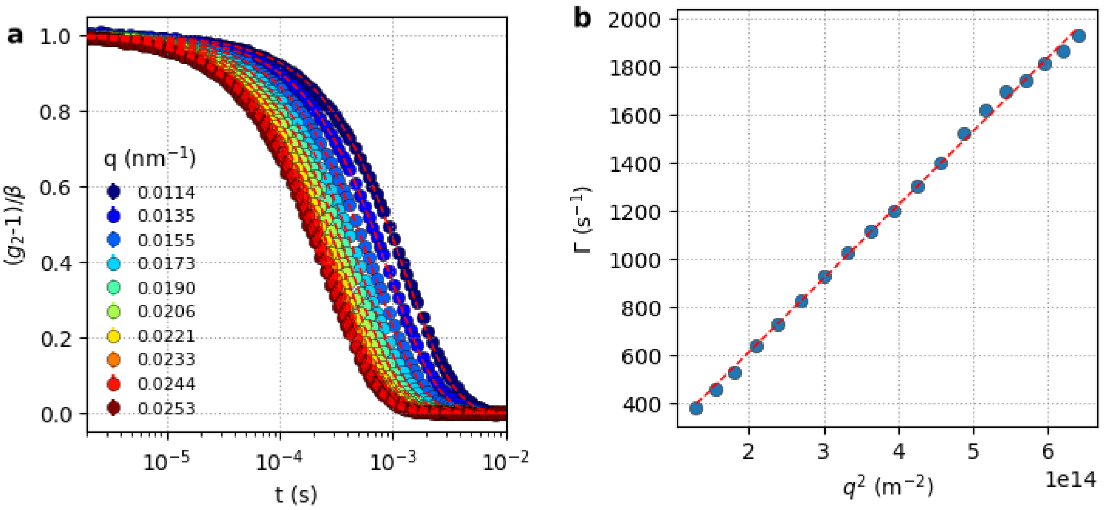

Fig. S6 | Dynamic light scattering (DLS) measurements of ferritin solutions. The data shown correspond to dilute condition ($c = 9$ mg/ml) in water-glycerol (with glycerol volume fraction $\nu_{\text{glyc}} = 0.55$) (a) The intensity autocorrelation functions, $g_2(q,t)$, is shown for different q values, as indicated in the legend. The $g_2(q,t)$ is normalised by the corresponding speckle contrast, β. (b) The extracted decorrelation rate, $\Gamma(q)$, obtained from the $g_2(q,t)$ using a single exponential fit. The solid line is a linear fit and the slope gives the diffusion coefficient D_0 in the dilute limit.

5 $H(q)$ model fit

To fit the experimental $D(q)S(q)/D_0$ data, the H(q) model was scaled with a concentration-dependent factor. The result of the fit is shown in Fig.S7.

The rescaling factor is present due to the influence of direct protein interactions on the diffusion. In more detail, since for this system $D^{long}(q)$ and $D^{short}(q)$ have the same q-dependence, we can combine Eq. 2 and Eq. 5 of the main paper so that:

$$D^{long}(q) = \frac{D^{short}(q)}{D_0} \cdot D_s^{direct} = \frac{H(q)}{S(q)} D_s^{direct} \tag{S1}$$

Therefore, for the $H(q)$ model to be able to describe the long-time diffusion, an additional factor has to be taken into account. To verify the validity of Eq. S1, we compare the experimental $D^{long}(q)/D^{short} = D_2(q)/D_1(q) = 0.12 \pm 0.04$ obtained from the two exponentials fit for concentration 730 mg/ml with the expected value of $D_s^{direct}/D_0 = 1/(1 + 2\chi\phi_h) = 0.13$, which shows good agreement.

At low protein concentration ($c = 70$ mg/ml) the correlation functions g_2 show Brownian motion ($\alpha \approx 1$) suggesting that the short-time diffusion is the main contribution on the observed dynamics. At

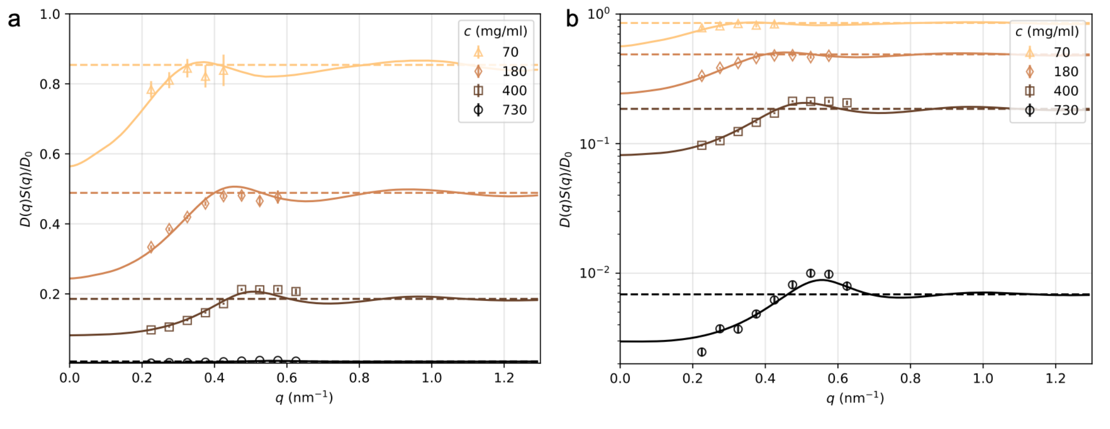

Fig. S7 | The experimental $D(q)S(q)/D_0$ as a function of momentum transfer q (empty symbols) and $H(q)$ model results using the $\delta\gamma$-theory (solid lines). The data points and lines are the same as in Fig. 5a, but the q range was extended to show the oscillations of the hydrodynamic function around the estimated D_s/D_0. The figure is shown in a) linear scale and in b) log scale to be able to see the data points for the highest protein concentration.

intermediate protein concentrations (c = 180-400 mg/ml), anomalous diffusion is evident with $\alpha < 1$, due to contributions from both short-time and long-time diffusion. As a consequence, the resulting diffusion coefficient and hydrodynamic functions shown in Figs. 3 and 4 (main paper) contain contributions from both short-time and long-time diffusion. Since the value of D_s/D_0 for these concentrations seem to follow more closely the long-time diffusion equation, we assume that the contribution of the long-time diffusion is dominating the signal. To understand the contribution of long-time diffusion in the signal at the various protein concentrations measured, we compare the scaling factor, hence the ratio of the experimental D_s over the theoretical value of D_s^{short} (Eq.15 of the main paper) and compare it with the theoretical value of D_s^{direct}/D_0. Figure S8 shows that for the lowest concentration (70 mg/ml) no correction factor is needed, consistent with the notion that we are probing only the short-time diffusion. For the intermediate concentrations (c = 180-400 mg/ml), the values are consistent with the model. For the highest concentrations (730 mg/ml), there is a deviation, possibly because the model of D_s^{short} is valid only for volume fractions up to ϕ = 0.45 according to Ref.[3].

6 Contrast evaluation

The values of the contrast $\beta(q)$ were estimated by using the equation [1, 2]:

$$\beta(q) = \beta_l(q)\beta_t \tag{S2}$$

The value of the transverse coherence was found to be $\beta_t \approx 0.5$ in various XFELs including the EuXFEL [1, 2, 4]. The longitudinal coherence, $\beta_l(q)$, describes the loss of coherence due to the finite bandwidth $\Delta E/E$, the geometry and speckle shape factor. $\beta_l(q) = (M_{\text{rad}} M_{\text{det}})^{-1}$ contains two contributions M_{rad} and M_{det}, which are:

$$M_{\text{rad}} = \sqrt{1 + \frac{q^2 \left(\frac{\Delta E}{E}\right)^2 \left[b_s^2 \cos^2(\theta) + t^2 \sin^2(\theta)\right]}{4\pi^2}} \tag{S3}$$

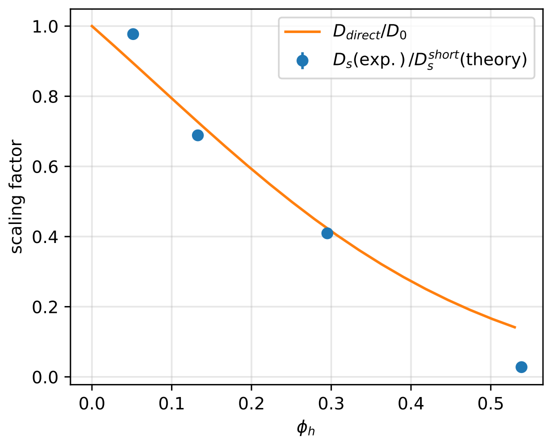

Fig. S8 | The scaling factor (blue circles) relates to the ratio of the experimental D_s over the theoretical short-time self-diffusion coefficient. The orange solid line represents the value of $D_s^{direct}/D_0 = 1/(1+2\chi\phi_h)$ where χ the contact value of the pair-correlation function was estimated approximating the protein interactions as hard spheres ($\chi = (1+0.5\phi)/(1-\phi)^2$).

$$M_{\text{det}} = \sqrt{1 + \frac{p^4 b_s^2 \left[b_s^2 \cos^2(2\theta) + t^2 \sin^2(2\theta)\right]}{\lambda^4 L^4 M_{\text{rad}}^2}}. \tag{S4}$$

With the constant b_s being the beam size, $\Delta E/E$ being the bandwidth, 2θ being the scattering angle, λ being the wavelength of the x-ray beam, L being the sample-detector distance, p being the pixel size, t being the sample thickness. The experimental parameters in the fit were fixed, except for the beam size b_s which effectively accounts for small changes in the experimental parameter between measurements. The value of the beam size was optimized for each sample in the fit g_2 function. The corresponding extracted values of the contrast with are reported in Fig.S9 and their respective b_s in Table S4.

Table S4 | Beam size obtained from the fit of $g_2(q,t)$ from Fig. 3 of the main manuscript.

c (mg/ml)	b_s (µm)
70	13.2 ± 1.5
180	12.8 ± 0.4
400	12.4 ± 0.3
730	13.4 ± 0.4

To independently verify the speckle contrast values obtained by the fit, we perform a single shot X-ray speckle visibility analysis (XSVS). Here, the contrast β can be obtained by analysing the properties of scattering intensity distribution $P(k,\bar{k},M)$ of a single frame [5]. The analytical form of $P(k,\bar{k},M)$ is:

$$P(k,\bar{k},M) = \frac{\Gamma(k+M)}{\Gamma(M)\Gamma(k+1)} \left(1 + \frac{M}{\bar{k}}\right)^{-k} \left(1 + \frac{\bar{k}}{M}\right)^{-M} \tag{S5}$$

where k is the photon count of each pixel per shot, $\bar{k}$ is the average number of photons per pixel per shot and M is the number of modes, which is related to the speckle contrast by $\beta = 1/M$. The contrast was estimated with two different methods: from the fit of the probability of finding a one- and two-photon events on a pixel $p_2 = P(k=2,\bar{k},M)$ and from a contrast estimator formula. The analytical form for p_1

and p_2 can be derived from Eq. S5, and is:

$$p_1 = P(k=1,\bar{k},M) = M\left(1+\frac{M}{\bar{k}}\right)^{-1}\left(1+\frac{\bar{k}}{M}\right)^{-M}, \quad \text{(S6)}$$

$$p_2 = P(k=2,\bar{k},M) = \frac{M(M+1)}{2}\left(1+\frac{M}{\bar{k}}\right)^{-2}\left(1+\frac{\bar{k}}{M}\right)^{-M}. \quad \text{(S7)}$$

From the analytical expression of p_1 and p_2, and defining R_{12} as $R_{12} = p_2/p_1$, β can be obtained with the following estimator[6]:

$$\beta = \frac{2\cdot R_{12} - \bar{k}}{\bar{k}(1-2\cdot R_{12})} \quad \text{(S8)}$$

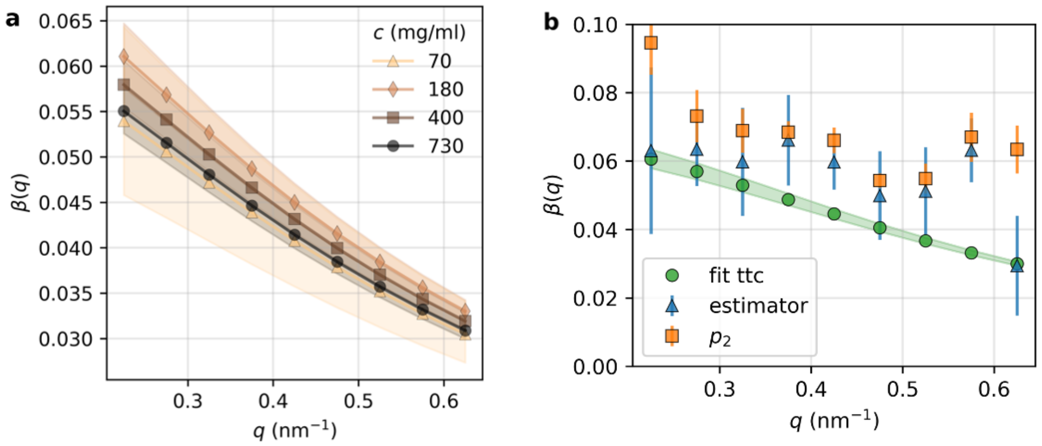

Fig. S9 | a) Speckle contrast β versus momentum transfer q, obtained after the optimization of the beam size in the fit of $g_2(q,t)$ from Fig. 3 of the main manuscript. The shaded area represents the standard error due to the uncertainties of the fit. b) Contrast estimation with three different methods for the data of protein concentration 180 mg/ml. The three methods correspond to: the fit results from the $g_2(q,t)$ functions (green circles and shaded area to indicate the standard error), the estimator of eq. S8 , the fit of the probability of two photon events given in Eq. S7 (orange squares).

The contrast values were obtained using the single images of pulses from 1 to 120 and the exact same bunch trains used for the calculation of $g_2(q,t)$. We observe that the contrast values obtained with different methods are overall consistent with each other. The minor discrepancies between the contrast obtained from the fit of the TTCs and from the XSVS analysis can be due to several reasons: First, in the XSVS analysis it is not straightforward to apply a cross-correlation correction [7] which corrects for the spurious correlations present due to the noise in the detector. Second, in the single image XSVS analysis a baseline correction is not trivial as it is for the two-time correlation functions, where the correlation of images at large time difference can be used as baseline. For XSVS, the large error bars are due to the very low counts which makes the baseline estimation challenging [8].

Mean square displacement and intermediate scattering function

The intensity autocorrelation function and the intermediate scattering function are related to both the mean square displacement of the particle and the time dependent concentration fluctuations. The width function, defined as

$$W(q,t) = -\frac{\ln[f(q,t)]}{q^2}, \tag{S9}$$

corresponds to the mean square displacement $\langle r^2(t)\rangle = W(q,t)$ in the case of a dilute system. In case of interacting particles this relation does not hold, as the mean-square displacement refers only to the self-part of the intermediate scattering function. In other words, $\langle r^2(t)\rangle$ includes only self-diffusion and not the contribution of collective diffusion. In case of interacting particles, only in the limit of $q \gg q_0$ the intermediate scattering function does not contain contributions from the collective diffusion, and therefore Eq. S9 holds.

For low protein concentration the relation between the self-diffusion coefficient and the mean square displacement is

$$\langle x^2(t)\rangle = 6D_s t. \tag{S10}$$

The presence of two exponential decays indicates that in the mean square displacement is not showing a simple relation as seen in Eq. S10. Instead, two different slopes are present, one D_s^{short} corresponding to time $t \ll \tau_i$ and the other one D_s^{long} at $t \gg \tau_i$.

Supplementary References

1. Reiser, M. *et al.* Resolving molecular diffusion and aggregation of antibody proteins with megahertz X-ray free-electron laser pulses. *Nat. Commun.* **13,** 5528 (2022).
2. Lehmkühler, F. *et al.* Emergence of Anomalous Dynamics in Soft Matter Probed at the European XFEL. *Proc. Natl. Acad. Sci. U.S.A.* **117,** 24110–24116 (2020).
3. Beenakker, C. Self-diffusion of spheres in a concentrated suspension. *Physica A* **120,** 388–410 (1983).
4. Madsen, A. *et al.* Materials Imaging and Dynamics (MID) instrument at the European X-ray Free-Electron Laser Facility. *J. Synchrotron Radiat.* **28,** 637–649 (2021).
5. Hruszkewycz, S. O. *et al.* High Contrast X-ray Speckle from Atomic-Scale Order in Liquids and Glasses. *Phys. Rev. Lett.* **109,** 185502 (2012).
6. Perakis, F. *et al.* Coherent X-rays reveal the influence of cage effects on ultrafast water dynamics. en. *Nat. Commun.* **9,** 1917 (2018).
7. Dallari, F. *et al.* Analysis Strategies for MHz XPCS at the European XFEL. *Appl. Sci.* **11,** 8037 (2021).
8. Möller, J. *et al.* Using low dose X-ray Speckle Visibility Spectroscopy to study dynamics of soft matter samples. *New J. Phys.* **23,** 093041 (2021).
