# Supplementary material for: Coherent X-rays reveal anomalous molecular diffusion and cage effects in crowded protein solutions
Source: Nat Commun. 2025 Nov 29;16:10814. doi: 10.1038/s41467-025-66972-6 (PMC12669764; doi:10.1038/s41467-025-66972-6)
Supplement: Supplementary file 2 — Reporting Summary [file 41467_2025_66972_MOESM2_ESM.pdf]

nature portfolio

Corresponding author(s): Fivos Perakis
Anita Girelli

Last updated by author(s): Mar 26, 2025

Reporting Summary

Nature Portfolio wishes to improve the reproducibility of the work that we publish. This form provides structure for consistency and transparency in reporting. For further information on Nature Portfolio policies, see our Editorial Policies and the Editorial Policy Checklist.

Statistics

For all statistical analyses, confirm that the following items are present in the figure legend, table legend, main text, or Methods section.

n/a	Confirmed	
☐	☒	The exact sample size (*n*) for each experimental group/condition, given as a discrete number and unit of measurement
☐	☒	A statement on whether measurements were taken from distinct samples or whether the same sample was measured repeatedly
☒	☐	The statistical test(s) used AND whether they are one- or two-sided<br>*Only common tests should be described solely by name; describe more complex techniques in the Methods section.*
☒	☐	A description of all covariates tested
☒	☐	A description of any assumptions or corrections, such as tests of normality and adjustment for multiple comparisons
☐	☒	A full description of the statistical parameters including central tendency (e.g. means) or other basic estimates (e.g. regression coefficient) AND variation (e.g. standard deviation) or associated estimates of uncertainty (e.g. confidence intervals)
☒	☐	For null hypothesis testing, the test statistic (e.g. *F*, *t*, *r*) with confidence intervals, effect sizes, degrees of freedom and *P* value noted<br>*Give P values as exact values whenever suitable.*
☒	☐	For Bayesian analysis, information on the choice of priors and Markov chain Monte Carlo settings
☒	☐	For hierarchical and complex designs, identification of the appropriate level for tests and full reporting of outcomes
☒	☐	Estimates of effect sizes (e.g. Cohen's *d*, Pearson's *r*), indicating how they were calculated

Our web collection on statistics for biologists contains articles on many of the points above.

Software and code

Policy information about availability of computer code

Data collection	Data recorded for the experiment at the European XFEL are available at [doi:10.22003/XFEL.EU-DATA-003094-00] and [doi:10.22003/XFEL.EU-DATA-005397-00]. Data recorded at ESRF are available at [doi:10.15151/ESRF-ES-1435809276]
Data analysis	The TTCs functions were extracted with the MID data processing pipeline embedded to the DAMNIT tool of the EuXFEL data analysis group (https://damnit.readthedocs.io/en/latest/).The code used to fit the data in this study is available from the corresponding authors upon request.

For manuscripts utilizing custom algorithms or software that are central to the research but not yet described in published literature, software must be made available to editors and reviewers. We strongly encourage code deposition in a community repository (e.g. GitHub). See the Nature Portfolio guidelines for submitting code & software for further information.

Data

Policy information about availability of data

All manuscripts must include a data availability statement. This statement should provide the following information, where applicable:

- Accession codes, unique identifiers, or web links for publicly available datasets
- A description of any restrictions on data availability
- For clinical datasets or third party data, please ensure that the statement adheres to our policy

Data recorded for the experiment at the European XFEL are available at [doi:10.22003/XFEL.EU-DATA-003094-00] and [doi:10.22003/XFEL.EU-DATA-005397-00]. Data recorded at ESRF are available at [doi:10.15151/ESRF-ES-1435809276]

Research involving human participants, their data, or biological material

Policy information about studies with human participants or human data. See also policy information about sex, gender (identity/presentation), and sexual orientation and race, ethnicity and racism.

Reporting on sex and gender	not relevant
Reporting on race, ethnicity, or other socially relevant groupings	not relevant
Population characteristics	not relevant
Recruitment	not relevant
Ethics oversight	not relevant

Note that full information on the approval of the study protocol must also be provided in the manuscript.

Field-specific reporting

Please select the one below that is the best fit for your research. If you are not sure, read the appropriate sections before making your selection.

☒ Life sciences ☐ Behavioural & social sciences ☐ Ecological, evolutionary & environmental sciences

For a reference copy of the document with all sections, see nature.com/documents/nr-reporting-summary-flat.pdf

Life sciences study design

All studies must disclose on these points even when the disclosure is negative.

Sample size	The measurements were repeated to increase the signal to noise ratio. This also ensured that the sample was measured in different position along the capillary to take into account of local variation, which were not seen. The total repetition number for the data shown in the manuscript is 6000 for each protein concentration for an X-ray transmission of 5.35x106
Data exclusions	none
Replication	The data were collected in 2 separate experiments
Randomization	no randomization was performed
Blinding	no blinding was performed

Reporting for specific materials, systems and methods

We require information from authors about some types of materials, experimental systems and methods used in many studies. Here, indicate whether each material, system or method listed is relevant to your study. If you are not sure if a list item applies to your research, read the appropriate section before selecting a response.

Materials & experimental systems

n/a	Involved in the study
☒	☐ Antibodies
☒	☐ Eukaryotic cell lines
☒	☐ Palaeontology and archaeology
☒	☐ Animals and other organisms
☒	☐ Clinical data
☒	☐ Dual use research of concern
☐	☐ Plants

Methods

n/a	Involved in the study
☒	☐ ChIP-seq
☒	☐ Flow cytometry
☒	☐ MRI-based neuroimaging

Plants

Seed stocks	not relevant
Novel plant genotypes	not relevant
Authentication	not relevant
